# Editorial: Cellular immune response and escape mechanisms of intracellular parasites

**DOI:** 10.3389/fimmu.2024.1362719

**Published:** 2024-01-12

**Authors:** Patrícia Maria Lourenço Dutra, Luciana Silva Rodrigues, Roberta Olmo Pinheiro, Wânia Ferraz Pereira Manfro, Igor Cestari, Sílvia Amaral Gonçalves Da-Silva

**Affiliations:** ^1^ Departamento de Microbiologia, Imunologia e Parasitologia, Faculdade de Ciências Médicas (FCM), Universidade do Estado do Rio de Janeiro (UERJ), Rio de Janeiro, Brazil; ^2^ Laboratório de Imunopatologia, Faculdade de Ciências Médicas (FCM), Universidade do Estado do Rio de Janeiro (UERJ), Rio de Janeiro, Brazil; ^3^ Laboratório de Hanseníase, Instituto Oswaldo Cruz, Fundação Oswaldo Cruz, Rio de Janeiro, Brazil; ^4^ Institute of Parasitology, McGill University, Ste Anne de Bellevue, QC, Canada; ^5^ Division of Experimental Medicine, McGill University, Montreal, QC, Canada

**Keywords:** cellular immune response, escape mechanisms, intracellular parasites, infectious disease, immunopathogenesis

Infectious parasitic diseases are among the most prevalent diseases in the world. Some of them are among the biggest causes of death in the world, presenting relevant negative consequences for global public health. The immune response is the first form of protection against these diseases and its effectiveness and magnitude can be influenced by numerous factors, such as immunocompetence, in the case of hosts, and virulence, in the case of parasites. Recently, innate immune pathways have gained more attention in studies focusing on the comprehension of immunopathogenesis of infectious diseases and there is an increased consensus that both innate and adaptive pathways act together to maintain homeostasis.

Macrophages show high plasticity and tissular macrophages are a very heterogeneous population. In didactic terms, macrophages can be classified as M1 or M2 cells. Classically activated macrophages (M1) produce inflammatory cytokines and nitric oxide (NO), which is associated with the control of the parasite burden. On the other hand, alternatively activated macrophages (M2) expressing arginase and producing polyamines are permissive to parasite replication. Cytokines and growth factors may modulate macrophage phenotype as well as efferocytosis. Vellozo et al. reviewed the protective role of M1 macrophages during experimental infection with *Trypanosoma cruzi* and discussed regulatory mechanisms that can control the cellular immune response, avoiding host tissue damage. Previous results demonstrated that T cells undergo activation-induced cell death during acute *T. cruzi* infection and that efferocytosis induces TGF-β and PGE-2 production, exacerbating *T. cruzi* replication. In contrast, inhibition of apoptosis *in vivo* using anti-FasL or a pan-caspase inhibitor increased cytokine production and improved the ability of macrophages to control parasite replication.

Although innate immune pathways contribute to the initial outcomes of infection, they might not be sufficient for host protection from infection. Benevides et al. demonstrated that mice deficient in the expression of a transcription factor that plays a critical role in regulating the function of lymphocytes (Blimp-1), produced higher expression of iNOS than wild-type mice; in contrast, the deficient mice presented higher parasitemia and mortality than wildtype mice. The_[IC1] [UdW2]_ authors have also demonstrated that the deficiency of Blimp-1 in T cells from the mice, did not impair an effective Th1 response during *T. cruzi* infection, although the mice presented a failure in the activation of CD8+ T cell responses, which are pivotal for restricting parasite growth. In addition, the authors discussed that Blimp-1 prevents the recruitment and activation of inflammatory monocytes and the subsequent release of inflammatory mediators such as TNF and NO, which cause liver damage and dysfunction.

Taken together, these data reinforce the hypothesis that host pathogen interaction modulates several pathways and the effective control of the infection depends on a balance between pro- and anti-inflammatory molecules. Excessive inflammatory stimulus may contribute to control infection, but, at the same time, may be deleterious for host cells.

Leprosy is an excellent model to study the balance between immunopathogenesis and host cell protection. The interaction between the innate and adaptive immune response, environmental factors and genetic predisposition may be responsible for the diverse clinical presentations of the disease. Pure neural leprosy (PNL) is a rare form of the disease where only neurological deficits manifest, without skin lesions. Pitta et al. described the serum cytokine profile during PNL and discussed the fact that *M. leprae* antigens are confined within the Schwann cell in the peripheral nerve and this may explain why the cytokines usually related to leprosy and leprosy reactions are not found in high levels in the serum of these patients. Increased MCP-1/CCL-2 and IP-10/CXCL-10 levels were observed and associated with nerve damage. PNL is an unconventional clinical form of the disease that should not be classified by the criteria used for classification of the dermatological presentations, but the data presented in this study, although with some limitations, may provide new information and raise new questions about the immunopathogenesis of PNL.

Another cytokine increased in PNL serum samples was IL-10. IL-10 is an immunoregulatory cytokine that may be associated with regulatory T cell (Treg) activation and function. Tarique et al. investigated the role of PD-1 pathway in leprosy. Although previous studies have demonstrated increased expression of PD-1 and PD-L1 in immune cells from leprosy patients, the role of PD-1 in damping the host effector Tregs cell response in human leprosy was not fully understood. In this scenario, authors evaluated the role of PD-1 in Tregs mediated suppression of T effector cells using the PD-1 blockade on FoxP3+ Tregs *in vitro*. In addition, they also studied the correlation between PD-1 expression and bacteriological index (BI). They demonstrated that the blockade of PD-1 restores Tregs mediated suppression of effector T cells and increased IL-10 secretion. In contrast, overexpression of PD-1 was associated with increased BI. Together, the data obtained suggest that manipulation of PD-1 pathway may contribute to the modulation of regulatory milieu in the clinical spectrum of leprosy.

Another example of host pathogen interaction in a co-evolutionary arm whereas the success of the parasite is the result of its ability to evade these host immune responses while still utilizing the host’s resources occurs during the infection with the protozoan parasite *Cryptosporidium* sp. *Cryptosporidium* sp is an opportunistic intracellular pathogen of the intestinal epithelium, and an important agent of infectious diarrhea and diarrhea-related death worldwide, especially in individuals with AIDS and in children. Cells of the innate immune system induce long non-coding RNAs (lncRNAs) which may regulate the immune response. However, certain microorganisms can use lncRNAs to regulate or evade the host’s immune response. Graham et al. showed that U90926, a lncRNA from epithelial cells, can be positively modulated by the parasite, resulting in the suppression of transcription of epithelial defence genes involved in the control of *Cryptosporidium* infection. Interestingly, this modulation of U90926 was associated with *Cryptosporidium parvum* virus (CSpV1), a double-stranded RNA virus that the parasite carries, highlighting a new parasite evasion strategy.

The five manuscripts comprising the Research Topic *Cellular Immune Response and Escape Mechanisms of Intracellular Parasites* described different strategies used by infectious agents to utilize host resources and machinery for their benefit and contributed to elucidating more about host pathogen interaction regulatory pathways.

## Author contributions

PD: Writing – original draft, Writing – review & editing. LR: Writing – original draft, Writing – review & editing. RP: Writing – original draft, Writing – review & editing. WP: Writing – original draft, Writing – review & editing. IC: Writing – original draft, Writing – review & editing. SD: Writing – original draft, Writing – review & editing.

